# Upregulation of T Cell Receptor Signaling Pathway Components in Gestational Diabetes Mellitus Patients: Joint Analysis of mRNA and circRNA Expression Profiles

**DOI:** 10.3389/fendo.2021.774608

**Published:** 2022-01-03

**Authors:** Yan-ming Chen, Qiong Zhu, Jie Cai, Zhi-jia Zhao, Bin-bin Yao, Li-ming Zhou, Lin-dan Ji, Jin Xu

**Affiliations:** ^1^ Department of Science and Education, Affiliated People’s Hospital of Ningbo University, Ningbo, China; ^2^ Department of Preventive Medicine, School of Medicine, Ningbo University, Ningbo, China; ^3^ Department of Pediatrics, Affiliated People’s Hospital of Ningbo University, Ningbo, China; ^4^ Department of Reproductive Medicine, Ningbo Women and Children’s Hospital, Ningbo, China; ^5^ Department of Biochemistry, School of Medicine, Ningbo University, Ningbo, China; ^6^ Zhejiang Key Laboratory of Pathophysiology, School of Medicine, Ningbo University, Ningbo, China

**Keywords:** gestational diabetes mellitus, mRNA, circRNA, T cell receptor signaling pathway, microarray analysis

## Abstract

**Objective:**

Gestational diabetes mellitus (GDM) is one of the most common complications of pregnancy, and its pathogenesis is still unclear. Studies have shown that circular RNAs (circRNAs) can regulate blood glucose levels by targeting mRNAs, but the role of circRNAs in GDM is still unknown. Therefore, a joint microarray analysis of circRNAs and their target mRNAs in GDM patients and healthy pregnant women was carried out.

**Methods:**

In this study, microarray analyses of mRNA and circRNA in 6 GDM patients and 6 healthy controls were conducted to identify the differentially expressed mRNA and circRNA in GDM patients, and some of the discovered mRNAs and circRNAs were further validated in additional 56 samples by quantitative realtime PCR (qRT-PCR) and droplet digital PCR (ddPCR).

**Results:**

Gene ontology and pathway analyses showed that the differentially expressed genes were significantly enriched in T cell immune-related pathways. Cross matching of the differentially expressed mRNAs and circRNAs in the top 10 KEGG pathways identified 4 genes (*CBLB*, *ITPR3*, *NFKBIA*, and *ICAM1*) and 4 corresponding circRNAs (circ-CBLB, circ-ITPR3, circ-NFKBIA, and circ-ICAM1), and these candidates were subsequently verified in larger samples. These differentially expressed circRNAs and their linear transcript mRNAs were all related to the T cell receptor signaling pathway, and PCR results confirmed the initial microarray results. Moreover, circRNA/miRNA/mRNA interactions and circRNA-binding proteins were predicted, and circ-CBLB, circ-ITPR3, and circ-ICAM1 may serve as GDM-related miRNA sponges and regulate the expression of CBLB, ITPR3, NFKBIA, and ICAM1 in cellular immune pathways.

**Conclusion:**

Upregulation of T cell receptor signaling pathway components may represent the major pathological mechanism underlying GDM, thus providing a potential approach for the prevention and treatment of GDM.

## Introduction

Gestational diabetes mellitus (GDM) refers to varying degrees of impaired glucose tolerance that occur for the first time during pregnancy, and it is one of the most common complications of pregnancy (1). According to the latest global diabetes map released by the International Diabetes Federation, the worldwide prevalence of gestational hyperglycemia in 2019 was 15.8%, and GDM accounted for 83.6% of these cases (2). In China, with the gradual relaxation of the family planning policy, the continuous postponement of childbearing, and the increase in irregular lifestyles and incidences of overweight and obesity, the incidence of GDM has significantly increased in recent years. The latest epidemiological surveys show that the prevalence rate of GDM in China ranges from 17.6 to 18.3% (3, 4), which is considerably higher than the global average. In addition, the abnormal increase in blood glucose levels in GDM patients markedly increases the incidence of adverse pregnancy outcomes. A large number of studies have shown that GDM increases the risk of maternal miscarriage, cesarean section, postpartum hemorrhage, preeclampsia, neonatal macrosomia, infants larger than gestational age, and congenital malformations, and GDM also leads to an increased future risk of type 2 diabetes (T2D) and cardiovascular disease for mothers and offsprings (5).

Great efforts have been devoted to identifying the risk factors and pathological mechanisms underlying GDM (6). The well-known risk factors for GDM includes old age, race, T2D family history, past stillbirth, anemia, BMI >25, sedentary lifestyle, smoking, sugary drinks, and other dietary factors (7, 8). The pathological process of GDM is mainly related to increased peripheral insulin resistance, impaired glucose uptake, and the role of obesity-related inflammatory factors (1). At present, the focus of research on GDM has gradually shifted from metabolic disorder to placental dysfunction and immune system activation. However, the related genetic mechanism remains unclear (9, 10). In recent years, with advances in new-generation sequencing technologies, the number of studies predicting GDM biomarkers has been increasing, and most of these studies focus on the prediction of mRNAs, miRNAs, and lncRNAs (11), and few studies focus on the prediction of circRNAs (12). Nevertheless, the interactions of mRNAs and circRNAs from a transcriptomics perspective are still unknown.

Circular RNAs (circRNAs), covalently closed cyclic molecules characterized by a lack of 3’ and 5’ polar ends, are novel noncoding RNAs (ncRNAs) that have recently attracted considerable interest from researchers (13). In the past, circRNAs have been considered to be byproducts of RNA splicing errors and useless fragments, but now, they have been shown to be important regulators of gene expression in the pathophysiological processes of many diseases (14). Specifically, circRNAs can work as miRNA sponges, transcriptional regulators, protein-binding molecules, protein function enhancers, and protein scaffolds and can sometimes even be translated into functional proteins (7, 15). In addition, circRNAs are abundantly expressed in cells and tissues and have the characteristics of relative stability, long half-lives, tissue specificity, and high evolutionary conservation among species (16), suggesting that circRNAs might be new biomarkers for clinical applications.

Recently, an increasing number of studies have reported that circRNAs might play important roles in the regulation of blood glucose levels, for example, by regulating islet β cell proliferation and insulin secretion. Xu et al. showed that circRNA Cdr1as (also known as CIRS-7) has a strong effect on miR-7 sponge/inhibitor, and the Cdr1as/miR-7 pathway further promotes insulin production and secretion by regulating the insulin granule secretion target Myrip and enhancing the insulin transcription target Pax6 (17). A subsequent mouse study found that the expression of Cdr1as and another circRNA, circHIPK3, was significantly downregulated in the islets of diabetic db/db mice. Further analysis revealed that circHIPK3 acts by sequestering a group of microRNAs, namely, miR-124-3p and miR-338-3p, and by regulating the expression of key β-cell genes, especially Akt1 (18). A cohort study found that hsa_circ_0054633 was significantly overexpressed in the sera and placentas of GDM women in the second and third trimesters of pregnancy, and its expression level was positively correlated with postprandial blood glucose levels and glycosylated hemoglobin levels (19). Later, Zhao et al. found that hsa_circ_0054633 may regulate β cell metabolism and participate in the pathogenesis of GDM by affecting the cell cycle (20). Yang et al. found that hsa_circ_102893 was significantly downregulated in peripheral blood of GDM patients, which could bind to RNA binding protein EIF4A3 and regulate cell cycle and apoptosis process through TNF-α/NF-κB signaling pathway, thus affecting insulin secretion and β cell function in GDM patients (21, 22).

Inspired by these findings, we speculated that circRNAs might be potential regulators of GDM. In the present study, we used genome-wide microarrays to identify differentially expressed mRNAs and circRNAs between GDM patients and healthy controls. The joint analysis of circRNA and mRNA expression profiles may provide relevant information for understanding the potential pathological mechanisms underlying GDM and developing new mechanism-based diagnostics and therapies.

## Materials and Methods

### Participants and Sample Collection

The participants were recruited from the Affiliated People’s Hospital of Ningbo University from September 2018 to November 2019. The recruited participants met the following inclusion criteria: 20–35 years old, Han nationality, and with BMI <30. GDM was diagnosed by the one-step criteria suggested by the WHO: fasting glucose level ≥5.1 mmol/L or 1 h 75 g oral glucose tolerance test (OGTT) ≥10.0 mmol/L or 2 h OGTT ≥8.5 mmol/L. The control group matched the GDM group through a 1:1 pattern according to age ( ± 2). Participants who met the following exclusion criteria were excluded from both groups: (1) history of diabetes, (2) history of taking hypoglycemic drugs, (3) recent fever, infection and other acute inflammatory periods, (4) pregnancy hypertension, polycystic ovary syndrome, (5) chronic diseases (thyroid dysfunction, cardio-cerebrovascular diseases, malignant tumors, etc.), (6) multiple pregnancies, and (7) the use of assisting reproductive technology. Finally, 68 pregnant women, 34 GDM patients and 34 healthy controls, were recruited in the current study. Six pairs of samples were used for microarray analysis and the remaining 28 pairs were used for further validation. The protocol of this study was approved by the Medical Ethics Committee of Ningbo University, and all participants signed the informed consent form.

### RNA Preparation and Microarray Analysis

Aliquots of 2 ml of fresh peripheral blood were collected from each participant, and peripheral blood mononuclear cells (PBMCs) were isolated by Ficoll separation solution (Bioss, USA). TRIzol pyrolysis solution (Omega, USA) was added, and then, the samples were stored in the freezer at −80°C before use. An HP total RNA extraction kit (Omega, USA) was used to extract the total RNA according to the manufacturer’s instructions. The RNA quality was evaluated by a NanoDrop 2000 spectrophotometer on the basis of an optical density (OD) 260/280 ratio ≥1.8 and an OD260/230 ratio ≥2.0. RNA integrity was measured using an Agilent 2100 Bioanalyzer. The intensity of the 18S and 28S rRNA bands was examined by 1% formaldehyde denaturing gel electrophoresis. RNA samples with an RNA integrity number (RIN) of ≥7.0 and 28S/18S >1.5 were adjusted to the same concentration and then subjected to microarray analysis.

Microarray analysis, namely, RNA amplification, probe labeling, hybridization, and data extraction, was performed by CapitalBio Corporation. The CapitalBio mRNA Amplification and Labeling Kit (CapitalBio, China) and circRNA Amplification and Labeling Kit (CapitalBio, China) were used to amplify and transcribe mRNA, respectively, and the RNase R-enriched circRNA was used to generate fluorescent cRNA. The labeled cRNAs were hybridized onto the CapitalBio Technology Human mRNA Array V4 (CapitalBio, China) and CapitalBio Technology Human CircRNA Array v2.0 (CapitalBio, China). Subsequently, the arrays were scanned using the Agilent Scanner G2565CA (Agilent, USA). Acquired array images were analyzed with Agilent Feature Extraction (v10.7) software. Overall, 12 mRNA microarray chips (6 GDM patients and 6 healthy controls) and 12 circRNA microarray chips (6 GDM patients and 6 healthy controls) were analyzed in this study.

### Statistical Analysis of the Microarray Data

Data summarization, quality control, quantile normalization, and analysis of differentially expressed mRNAs/circRNAs were performed with GeneSpring v13.0 (Agilent, USA). Individually, mRNAs and circRNAs with a fold change ≥2 or ≤0.5 and FDR <0.05 were considered significantly differentially expressed. Gene annotation and biological interpretation of the identified differentially expressed mRNAs/circRNAs were performed using the Database for Annotation, Visualization, and Integrated Discovery (DAVID) v6.8. Biological functions, represented by the Gene Ontology terms (http://geneontology.org/) and the Kyoto Encyclopedia of Genes and Genomes pathways (http://genome.jp/kegg/), were considered significant at a Benjamini–Hochberg-corrected *P <*0.05.

### Prediction of circRNA/miRNA/mRNA Interactions and circRNA-Binding Proteins

The miRanda software and TargetScan database (http://www.targetscan.org/) were used to predict target miRNAs for candidate mRNAs and circRNAs. Cytoscape 3.2.1 software was used to construct a circRNA–miRNA–mRNA coexpression network to predict the functions and interactions between RNAs. In addition, the CircInteractome database (https://circinteractome.nia.nih.gov/) was used to predict the candidate binding proteins of circRNAs.

### Validation of Candidate mRNAs and circRNAs

Four differentially expressed mRNAs (CBLB, ITPR3, NFKBIA, and ICAM1) and four differentially expressed circRNAs (circ-CBLB, circ-ITPR3, circ-NFKBIA, and circ-ICAM1) selected from the T Cell Receptor Signaling Pathway were further validated by quantitative real-time PCR (qRT-PCR) and droplet digital PCR (ddPCR) in another 28 independent samples. qRT-PCR was performed with the LightCycler 480 SYBR Green I Master Mix (Roche, Germany), and ddPCR was performed by QX200™ ddPCR™ EvaGreenSupermix (Bio–Rad, USA) according to the manufacturer’s instructions. After qRT-PCR, the relative mRNA expression was calculated by the 2^−ΔΔCT^ method, with GAPDH as an internal control. The primers for candidate mRNAs and circRNAs are listed in **Supplemental Table 1**. The products of qPCR and ddPCR were further confirmed by electrophoresis.

### Statistical Analysis

SPSS 22.0 statistical software was used for statistical analysis. For the baseline information and clinical data of the participants, the continuous normal data are expressed as the mean ± standard deviation (*X* ± *S*), and the difference between groups were compared by two independent samples *t*-tests; the nonnormal continuous data are expressed as the median (upper and lower quartiles), and the Mann–Whitney rank test was used for intergroup comparison; the classified data are presented in the form of cases (%), and the intergroup comparisons were conducted by *χ^2^* test or Fisher exact probability method. The verification results of candidate circRNAs and mRNAs between the GDM group and the healthy pregnant group were expressed by $\bar{X}\pm S$ or median (upper and lower quartiles). Paired sample *t*-test or Wilcoxon signed rank test was used for inter-group comparison. For the microarray test results, the data are normalized and analyzed by Agilent GeneSpring software, and cluster analysis is carried out by Cluster 3.0 software. Statistical images were drawn by GraphPad Prism 7.0 and OriginPro 2019b drawing software. Statistical significance was defined as *P <*0.05.

## Results

### Characteristics of the Participants

In this study, 34 pairs of pregnant women in the GDM and control groups were selected. Of these pairs, samples from 6 pairs were randomly selected for mRNA/circRNA microarray analysis, and samples from the remaining 28 pairs were tested for follow-up verification. The epidemiological characteristics and clinical indicators of these 34 pairs of participants are shown in **Supplemental Table 2**. There was no significant difference in the general characteristic indices between the two groups, except for the significantly higher OGTT level in the GDM group; these results indicated that the baseline indices of the two groups were well matched.

### Identification of the Differentially Expressed mRNAs and circRNAs

The current microarray data are available in the Gene Expression Omnibus (GEO, accession number GSE182737). The cluster analysis showed that both the expression profiles of mRNAs and circRNAs could distinguish the GDM group from the control group (**Supplemental Figures 1A**, **2A**). A total of 641 differentially expressed mRNAs were identified between the GDM group and the control group. Among these mRNAs, 269 were upregulated and 372 were downregulated (**Supplemental Figure 1B**). In addition, there were 7,950 differentially expressed circRNAs, of which 3,414 were upregulated and 4,536 were downregulated (**Supplemental Figure 2B**). Scatter plots and volcano maps were made according to their signal values and differential distributions (**Supplemental Figures 1C–D**, **2C–D**). The top 10 mRNAs and circRNAs that were the most significantly upregulated and downregulated are shown in **Supplemental Tables 3** and **4**.

### Functional Annotation and Enrichment Analysis of the Differentially Expressed mRNAs/circRNAs

**Figure 1:**
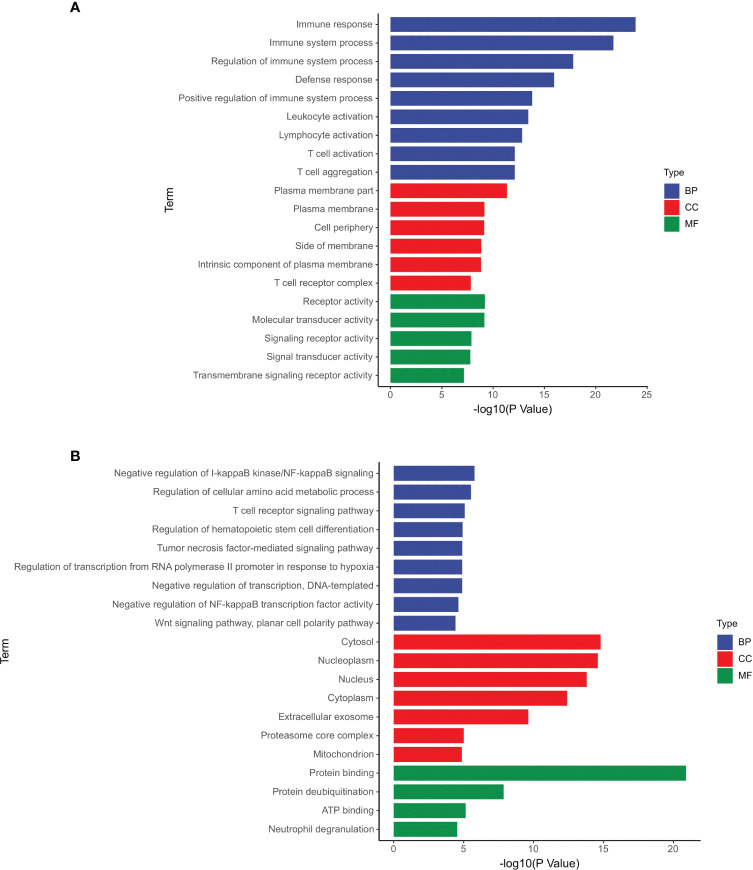
GO terms of the differentially expressed mRNAs and circRNAs. **(A)** Differentially expressed mRNAs; **(B)** differentially expressed circRNAs.

According to the GO analysis, the differentially expressed mRNAs and circRNAs were enriched in many similar GO terms as well as distinct GO terms (**Figure 1**). Compared with the profile of healthy controls, the GDM-associated differentially expressed mRNAs were primarily enriched in immune-related processes, such as immune response (GO:0006955), T cell aggregation (GO:0070489), and T cell activation (GO:0042110) (**Figure 1A**). Moreover, the GDM-associated differentially expressed circRNAs were enriched in the negative regulation of NF-kappaB signaling (GO:0043124), regulation of cellular amino acid metabolic process (GO:0006521), and T cell receptor signaling pathway (GO:0050852) (**Figure 1B**). Therefore, the majority of differentially expressed genes appear to be associated with the immune response mediated by T cells and receptor activity.

**Figure 2:**
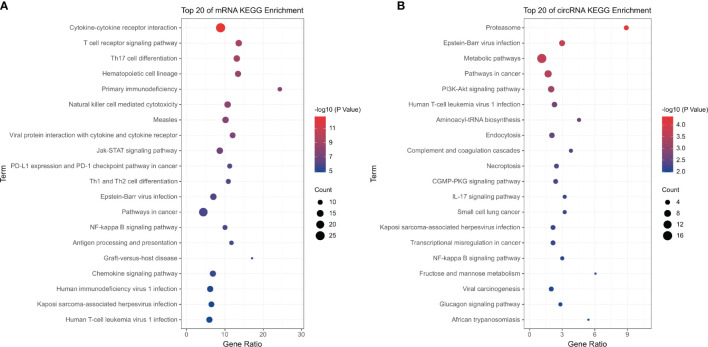
KEGG pathway analysis of the differentially expressed mRNAs and circRNAs. **(A)** Differentially expressed mRNAs; **(B)** differentially expressed circRNAs.

Similarly, KEGG pathway enrichment analysis showed that the significantly enriched pathways of differentially expressed mRNAs were immune-related pathways such as the T cell receptor signaling pathway (hsa04660), Th17 cell differentiation (hsa04659), primary immunodeficiency (hsa05340), and natural killer cell-mediated cytotoxicity (hsa04650) (**Figure 2A**). The pathways enriched by differentially expressed circRNAs were inflammatory and immune-related pathways such as proteasome (hsa03050), Epstein–Barr virus infection (hsa05169), human T-cell leukemia virus-1 infection (hsa05166), and IL-17 signaling pathways (hsa04657) (**Figure 2B**).

Further analysis indicated that the upregulated mRNAs were significantly enriched in pathways that were consistent with the overall result, which were immune-related pathways, especially T cell-related immunity (**Supplemental Figure 3A**). The pathways enriched by differentially expressed downregulated mRNAs were infection-related pathways, such as cytokine–cytokine receptor interaction (hsa04060), influenza A (hsa05164), hepatitis C (hsa05160), and human cytomegalovirus infection (hsa05163) (**Supplemental Figure 3B**).

### Validation of Candidate mRNAs and circRNAs

Cross matching of the differentially expressed mRNAs and circRNAs in the top 10 KEGG pathways, 4 genes (*CBLB*, *ITPR3*, *NFKBIA*, and *ICAM1*) and 4 corresponding circRNAs (circ-CBLB, circ-ITPR3, circ-NFKBIA, and circ-ICAM1) were identified. These mRNAs and circRNAs are all listed in the T cell receptor signaling pathway. The results of the original microarray expression of these candidate mRNAs and circRNAs are shown in **Supplemental Table 5**.

**Figure 3:**
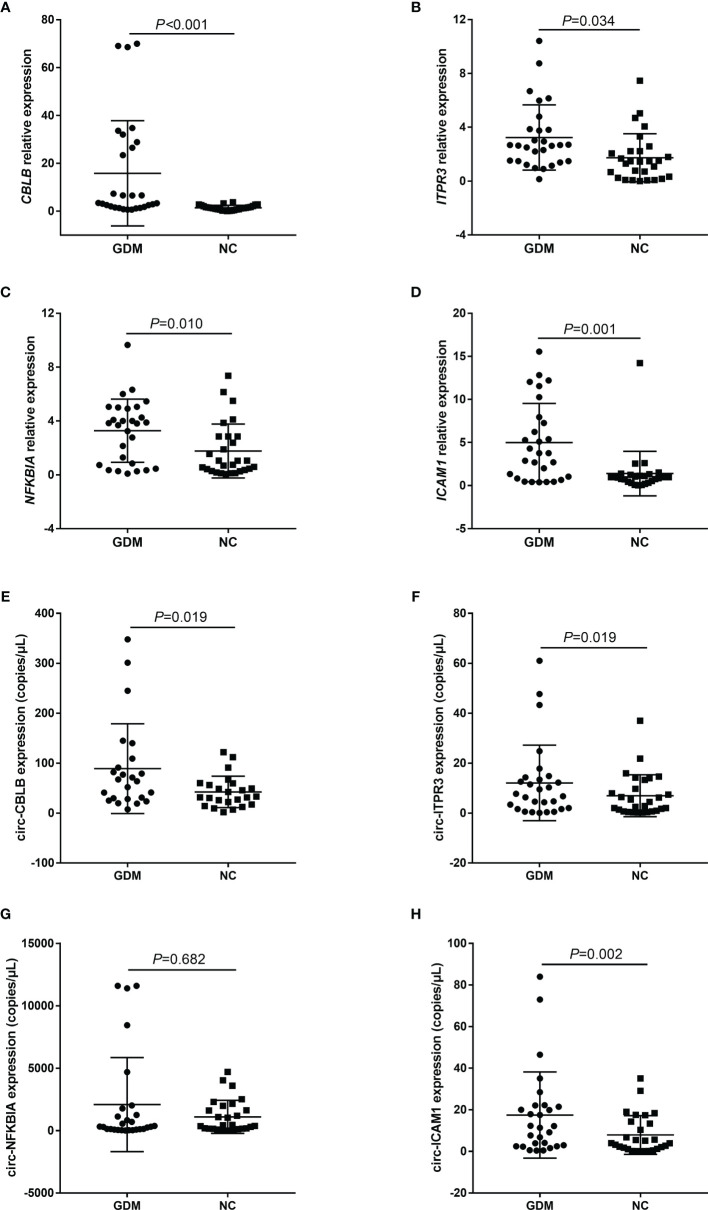
Microarray data were validated by qRT-PCR and ddPCR. **(A–H)** represent the expression distribution of CBLB, ITPR3, NFKBIA, and ICAM1 and their corresponding candidate circRNAs, circ-CBLB, circ-ITPR3, circ-NFKBIA, and circ-ICAM1, in different samples of the GDM group and control group, respectively. NC, Normal Control.

In this study, the expression levels of candidate mRNAs and circRNAs were verified by qRT-PCR and ddPCR in the 56 samples. The results indicated that the above four mRNAs and three circRNAs (circ-CBLB, circ-ITPR3, and circ-ICAM1) were significantly highly expressed in the GDM group, which was consistent with our microarray results (**Figure 3**).

### Prediction of circRNA/miRNA/mRNA Interactions and circRNA-Binding Proteins

A competing endogenous RNA (ceRNA) regulatory network was constructed by integrating the correlations and regulatory relationships among circRNAs, miRNAs, and mRNAs (**Supplemental Figure 4**). There are two regulatory relationships of circRNA–miRNA and miRNA–mRNA in the network, thus forming a circRNA–miRNA–mRNA coexpression model. The network contains 4 significantly upregulated candidate circRNAs, 4 significantly upregulated candidate mRNAs, and 594 predicted miRNAs. By calculating the degrees of each node in the network, we found that the nodes with the most degrees in the three kinds of RNA were circ-ITPR3, CBLB, and hsa-miR-203a-3p. In addition, the circRNA–miRNA–mRNA coexpression model showed that the network predicted a total of 16 key miRNAs that may play an intermediate regulatory role.

The binding proteins of the differentially expressed circRNAs were also predicted. The results indicated that circ-ITPR3 had the most abundant RNA-binding protein sites, and the largest number of RNA-binding proteins bound to circ-ITPR3 was EIF4A3, with 58 sites, followed by FMRP, IGF2BP, AGO2, and HuR. In addition, EIF4A3 bound to the flanking regions of all three candidate circRNAs (**Supplemental Figure 5**).

## Discussion

GDM is the most common complication of pregnancy, affecting up to 20% of pregnancies; GDM significantly contributes to preeclampsia and depression, and requiring a caesarean section (23). Because complex pathways are implicated in the pathophysiology of GDM, there are still no specific means of predicting or preventing GDM progression. Gene expression microarrays have been widely used in diabetic studies because alterations in transcriptional profiles provide a robust and sensitive way to better understand the pathogenesis of the disease. mRNA and ncRNA microarray analyses have previously been used to investigate the mechanisms underlying diabetic neuropathy (24), diabetic nephropathy (25), diabetic cardiomyopathy (26), and diabetic periodontitis (27). Similarly, microarray analyses have also been conducted in GDM. Genomic expression profiles of blood and placenta revealed significant immune-related pathways in Chinese women with gestational diabetes mellitus (28). The lncRNA microarray analysis revealed that lncRNAs ERMP1, TSPAN32, and MRPL38 form a coexpression network with TPH1, which is mainly involved in the tryptophan metabolism pathway, and lncRNA RPL13P5 forms a coexpression network with the TSC2 gene *via* the pi3k-akt and insulin signaling pathways (29). The miRNA expression profiles of plasma samples revealed that miR-574-5p and miR-3135b may serve as metabolic regulators of glucose and lipid levels in GDM (30).

CircRNA expression profiles were also measured in patients with GDM. One study analyzed the circRNA expression profiles in umbilical cord blood exosomes from normal subjects and gestational diabetes mellitus patients, and these results may delineate the roles of exosomal circRNAs in GDM development and fetal growth (31). However, the results cannot be used as predictive biomarkers for GDM since umbilical cord blood was used. Another study measured circRNA expression profiling in 6 paired women (with and without GDM) and showed that hsa_circRNA_102893 may be a potential novel and stable noninvasive biomarker for detecting GDM in early pregnancy (32). In addition, Yan et al. conducted transcriptome sequencing of placental tissues in Chinese pregnant women, and found 482 differentially expressed circRNAs in GDM patients. These circRNAs were significantly enriched in pathways related to glycometabolism and lipometabolism processes (33). However, these studies only screened the differentially expressed circRNAs, no further mRNA–circRNA joint analysis was conducted, and no potential pathways were further examined. Therefore, performing new transcriptional microarray analyses with commonly used human sample material, such as PBMCs, to identify the gene expression signatures and potential predictive biomarkers of GDM is necessary. The current study revealed that differentially expressed mRNAs/circRNAs were mainly enriched in immune-related pathways, such as the T cell receptor signaling pathway, Th17 cell differentiation, primary immunodeficiency, and NK cell-mediated cytotoxicity, strongly suggesting that GDM may involve immune responses, especially responses associated with T cell immune regulation. Furthermore, cross matching of the differentially expressed mRNAs and circRNAs in the top 10 KEGG pathways identified 4 genes and 4 corresponding circRNAs, which were all listed in the T cell receptor signaling pathway.

GDM has been associated with an impaired maternal immune response (34). During normal pregnancy, the antigenic substances expressed by the fetus can trigger maternal immune activation to a certain extent, and then, T cells and NK cells can help maintain homeostasis (35). When metabolism and immunity in the pregnancy environment are severely challenged, the mother will superimpose the dual effects of low-grade systemic inflammation and insulin resistance, thereby promoting the occurrence of GDM (36). Studies have shown that in GDM patients, the proportion of activated CD4^+^ T cells significantly increased, while the percentage of CD8^+^ T cells decreased, suggesting a state of superactivation and a deficiency of suppressive mechanisms (9). Moreover, the percentage of Th17 cells and the ratio of proinflammatory cells (Th17:Treg, Th17.1:Treg, and Th1:Treg) were increased in GDM patients, which significantly promoted the secretion of proinflammatory factors, such as IL17 and TNF α (37). Abnormal elevation of TNF α can further interfere with insulin receptor tyrosine autophosphorylation and promote serine phosphorylation of insulin receptor substrate 1 (IRS1), thus destroying the insulin signal cascade (38, 39). Before our study, two independent microarray studies reported similar results (28, 40). A global placental gene expression study identified sixty-six genes participating in cell functions involving cell activation, immune response, organ development, and regulation of cell death that were differentially expressed in GDM placentas (40). Genomic expression profiles of blood and placenta reveal significant immune-related pathways, ‘natural killer cell mediated cytotoxicity’ in blood, and ‘cytokine–cytokine receptor interaction’ in placenta, in Chinese women with gestational diabetes mellitus (28). The current study not only revealed that upregulation of T cell receptor signaling pathway components might be the major pathological mechanism underlying GDM but also suggested that circRNAs circ-CBLB, circ-ITPR3, circ-ICAM1, and their related linear transcripts *CBLB*, *ITPR3*, *NFKBIA*, and *ICAM1* might be potential biomarkers for GDM.

Circ-CBLB is composed of 398 nt and is located on human chromosome 3, and its corresponding parent gene is *CBLB*, which encodes E3 ubiquitin protein ligase; this protein promotes ubiquitin-mediated protein degradation by transferring ubiquitin from E2 ubiquitin-binding enzyme to the matrix. Some studies have shown that CBLB regulates T cell tolerance in peripheral blood and plays a key role in host pathogen defense and antitumor immunity (41). In the process of T cell receptor signal transduction, CBLB can mediate the hydrolysis of the upstream proteins ZAP70 and LCK through the ubiquitin pathway, which is involved in the negative feedback regulation of T cell receptor signaling (42). Interestingly, our microarray results showed that the expression of *CBLB* significantly upregulated in the GDM group and that the expression of the upstream *ZAP70*, *LCK*, and CD3 family molecules (*CD3D*, *CD3E*, *CD3G*, and *CD247*) of the T cell receptor signaling pathway was also significantly upregulated (**Supplemental Figure 6**). The result was also verified by qRT-PCR and strongly suggests that T cell immunity in GDM patients may be in a state of hyperactivation. Hansen also found that the expression of *CBLB* was significantly upregulated in T2D patients (43). Further functional studies showed that the loss of specificity of *CBLB* led to insulin and glucose tolerance test response disorder (44), and blocking CBLB-induced macrophage activation could improve insulin resistance (45). The current study also found that circ-CBLB expression was significantly upregulated in GDM patients, which has not been previously reported in diabetes. Considering the transcriptional regulation of parental genes by circRNAs, circ-CBLB may affect the expression of *CBLB* by regulating the alternative splicing of corresponding mRNA transcripts. However, this possibility still requires further investigation.

Circ-ITPR3 is located on the positive chain of chromosome 6 and consists of 5146 nt. In the current study, it was predicted that miR-24-3p may bind to circ-ITPR3 and then affect the downstream target gene *NFKBIA*. miR-24-3p expression was found to be significantly downregulated in diabetes, and the expression level of miR-24-3p was significantly correlated with serum insulin and HbA1c levels (46). NFKBIA is a member of the NF-κB inhibitor family and can inhibit inflammation. T cell receptor activation and related TNF secretion can induce an increase in *NFKBIA* expression (47). Therefore, it can be inferred that the upregulation of circ-ITPR3 expression may significantly increase *NFKBIA* expression by inhibiting the expression of miR-24-3p, playing a negative feedback role in GDM-related inflammation. In addition, we found that the largest number of circ-ITPR3 binding site was EIF4A3, which is an ATP-dependent RNA helicase and widely involved in RNA splicing and nonsense-mediated mRNA decay. It was also reported that EIF4A3 can regulate cell cycle and apoptosis through TNF-α/NF-ĸB signaling pathway, which is one of the key signal pathways affecting diabetes (48, 49). Therefore, we speculate that circ-ITPR3 may regulate the expression of *ITPR3* and NF-ĸB family member *NFKBIA* by recruiting EIF4A3, which subsequently affects the development of GDM.

The parent gene of circ-ITPR3 is *ITPR3*, which encodes inositol 1,4,5-trisphosphate receptors (IP3R), a calcium release channel that responds to the second messenger inositol 1,4,5-trisphosphate (IP3). IP3 can be phosphorylated by inositol 1,4,5-trisphosphate 3-kinase and act as a negative regulator of Ca2+/nuclear factor of activated T cells (NFAT) in the T cell receptor signaling pathway (50). Upregulation of ITPR3 expression may induce autoimmune disorders and promote the occurrence and development of T1D by activating T cells (51). Moreover, genetic studies have shown that the SNPs of *ITPR3* are closely associated with T1D in Caucasian populations (52, 53) and autoimmune diseases (namely, systemic lupus erythematosus, rheumatoid arthritis, and Graves’ disease) in Japanese populations (54). Although the present study showed that *ITPR3* expression is significantly upregulated in the GDM group, its potential mechanism still needs to be further studied.

The corresponding parent gene of circ-ICAM1 is *ICAM1*, which encodes intercellular adhesion molecule 1, which is a glycoprotein on the surface of antigen-presenting cells. ICAM1 is an important part of T cell immune synapses and promotes the interaction of T cell receptor signal transduction molecules and the activation of the T cell receptor signaling pathway (55, 56). It has been reported that the plasma ICAM1 concentration of patients with T2D was significantly higher than that of control subjects (57). With the progression of diabetic complications, such as diabetic nephropathy (56), diabetic peripheral neuropathy (58), and diabetic retinopathy (59), the level of ICAM1 further increases. Upregulation of ICAM1 expression was also observed in GDM patients (60), which was consistent with the present result. The potential mechanism suggested that the increase in the ICAM1 levels may cause vascular endothelial dysfunction, leading to vascular inflammation, vascular functional imbalance, and increased oxidative stress, thus promoting the progression of hyperglycemia (61). Although the current study found that circ-ICAM1 expression was significantly upregulated in the GDM group, the target effect of circ-ICAM1 on diabetes-related diseases has never been reported. The constructed ceRNA network predicted that both miR-874-3p and miR-339-5p significantly interacted with circ-ICAM1. Huo et al. found that the expression of miR-874-3p in diabetic rats was significantly downregulated, which led to increased islet β cell apoptosis and diabetes-induced erectile dysfunction by targeting the inhibition of the nuclear protein 1 (Nupr1)-mediated pathway (62). It was also observed that miR-339-5p and miR-874-3p mediate high glucose-induced endothelial inflammation by targeting interleukin-1 receptor-associated kinase 1 (*IRAK1*) expression (63). The increased *IRAK1* expression was related to the upregulation of *ICAM1* gene expression and the enhancement of monocyte adhesion, which may be due to the weakening of the feedback inhibitory effect of IRAK1 on endothelial inflammation and the decrease in the anti-inflammatory effects of miR-339-5p and miR-874-3p in the diabetic environment (63). Therefore, the significant upregulation of *ICAM1* expression in the current study may be due to the miRNA sponge effect of circ-ICAM1 on miR-874-3p and miR-339-5p in GDM patients.

## Conclusion

**Figure 4:**
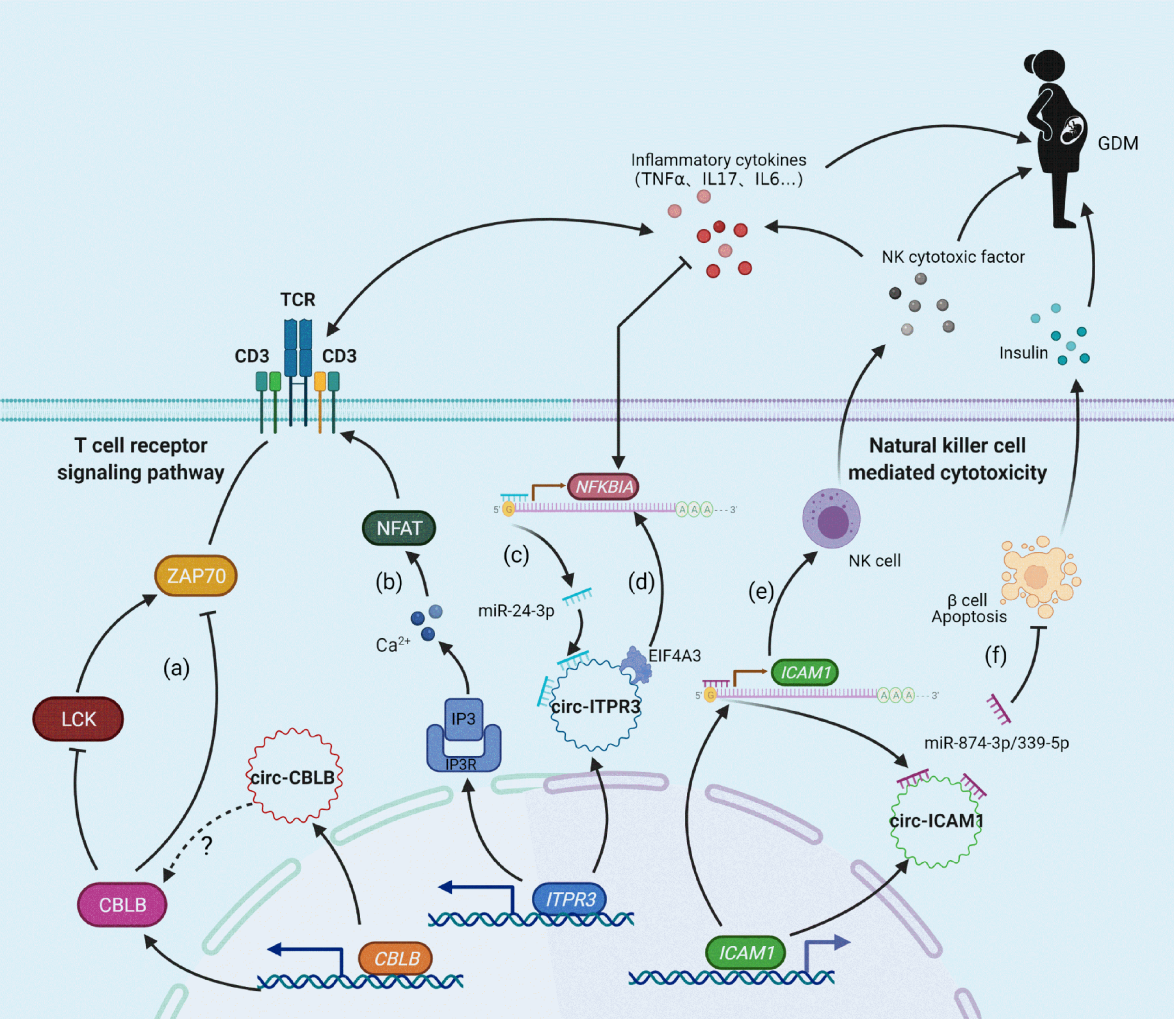
Molecular mechanism of the relationship between candidate circRNAs/mRNAs and GDM. **(A)** CBLB can inhibit LCK and ZAP70 expression levels by ubiquitination, thus exerting negative feedback regulation on T-cell receptor activation; **(B)** ITPR3 can encode IP3R in response to IP3-mediated Ca2^+^ release and promote the activation of the T cell receptor signaling pathway; **(C, E)** circ-ITPR3 and circ-ICAM1 act as miRNA sponges for miR-24-3p and miR-874-3p/miR-339-5p, respectively, which induces upregulation of the downstream target genes NFKBIA and ICAM1; **(D)** circ-ITPR3 regulates the expression of NFKBIA by recruiting EIF4A3. **(F)** The adsorption of circ-ICAM1 to miR-874-3p weakens the anti-apoptotic effect of miR-874-3p, which increases the apoptosis of β cells and decreases the release of insulin.

In summary, these findings provide the first demonstration that upregulation of T cell receptor signaling pathway components may be the major pathological mechanism underlying GDM. circ-CBLB, circ-ITPR3, and circ-ICAM1 may serve as GDM-related miRNA sponges and regulate the expression of CBLB, ITPR3, NFKBIA, and ICAM1 in cellular immune pathways, inducing vascular inflammation, immune activation, β cell apoptosis, and other related pathophysiological processes, finally promoting the occurrence and development of GDM (**Figure 4**). T cell receptor immune repertoire analysis is being carried out in GDM patients, which may provide more details. Moreover, functional analyses of the relevant circRNAs, miRNAs, mRNAs, and proteins are necessary, and these analyses may provide a potential approach for the prevention and treatment of GDM.

## Data Availability Statement

The datasets presented in this study can be found in online repositories. The names of the repository/repositories and accession number(s) can be found in the article/**Supplementary Material**.

## Ethics Statement

The studies involving human participants were reviewed and approved by the Medical Ethics Committee of Ningbo University. The patients/participants provided their written informed consent to participate in this study.

## Author Contributions

L-DJ and JX raised the idea for the study. Y-MC, QZ, JC, Z-JZ, and B-BY contributed to the study design, sample collection, and conducted the experiment. Y-MC, L-MZ, L-DJ, and JX analyzed the data and write the manuscript. All authors contributed to the article and approved the submitted version.

## Funding

This study was supported by the Zhejiang Public Welfare Technology Application Research Program (LGF20H260009, LGF20H040005), the Zhejiang Medical and Health Science and Technology Program (2019KY648), the Ningbo Nonprofit Science and Technology Project (2019C50097 and 2021S132), and the Ningbo Medical and Health Brand Discipline (PPXK2018-06).

## Conflict of Interest

The authors declare that the research was conducted in the absence of any commercial or financial relationships that could be construed as a potential conflict of interest.

## Publisher’s Note

All claims expressed in this article are solely those of the authors and do not necessarily represent those of their affiliated organizations, or those of the publisher, the editors and the reviewers. Any product that may be evaluated in this article, or claim that may be made by its manufacturer, is not guaranteed or endorsed by the publisher.

## Supplementary Material

Supplementary Figure 1 The expression profiles of mRNAs in the GDM and control groups. **(A)** cluster analysis of mRNA microarray. The red column at the bottom represents the GDM group, and the blue column represents the control group; red indicates relatively high expression, green indicates relatively low expression; **(B)** the number of differentially expressed mRNAs; **(C)** the scatter plot of mRNA signal value; **(D)** the volcano plot analysis of the differentially expressed mRNAs, in which red and green dots represent up- and downregulated mRNAs, respectively.

Supplementary Figure 2 The expression profiles of circRNAs in the GDM and control groups. **(A)** cluster analysis of circRNA microarray. The red column at the bottom represents the GDM group, and the blue column represents the control group; red indicates relatively high expression, green indicates relatively low expression; **(B)** the number of differentially expressed circRNAs; **(C)** the scatter plot of circRNA signal value; **(D)** the volcano plot analysis of the differentially expressed circRNAs, in which red and green dots represent up- and downregulated circRNAs, respectively.

Supplementary Figure 3 KEGG pathway analysis of the upregulated and downregulated mRNAs. **(A)** upregulated mRNAs; **(B)** downregulated mRNAs.

Supplementary Figure 4 circRNA-miRNA-mRNA coexpression network. The circular node represents the candidate circRNA, the square represents the candidate mRNA, and the triangle represents the predicted miRNA. With the number of nodes interacting with each other, that is, the number of degrees, the node changes from small to large. With the differential expression ratio of candidate RNAs between the two groups from low to high, the node color gradually changed from light red to deep red. The predicted miRNA has no expression value and is expressed as a green node.

Supplementary Figure 5 Prediction of candidate circRNA-binding protein sites. **(A–C)** represent the distribution of RNA-binding proteins and their binding sites of circ-ITPR3, circ-CBLB and circ-ICAM1, respectively.

Supplementary Figure 6 Microarray data of *ZAP70*, *LCK* and CD3 family molecules. The results are expressed as the mean ± SD The FC value of the control group was set as 1. *represents statistical significance (*P*<0.05) compared with the control group.
