# Supplementary material for: Upregulation of T Cell Receptor Signaling Pathway Components in Gestational Diabetes Mellitus Patients: Joint Analysis of mRNA and circRNA Expression Profiles
Source: Front Endocrinol (Lausanne). 2022 Jan 3;12:774608. doi: 10.3389/fendo.2021.774608 (PMC8763273; doi:10.3389/fendo.2021.774608)
Supplement: Supplementary file 7 [file DataSheet_1.docx]

Supplemental Table 1 Primers for candidate mRNAs/circRNAs

|  | Forward primer（5’-3’） | Reverse primer（5’-3’） |
| --- | --- | --- |
| *CBLB* | AATCCCCGAAAAGGTCGAATTT | CACAGTCTTACCACTTTGTCCAT |
| *ITPR3* | CCAAGCAGACTAAGCAGGACA | ACACTGCCATACTTCACGACA |
| *NFKBIA* | CTCCGAGACTTTCGAGGAAATAC | GCCATTGTAGTTGGTAGCCTTCA |
| *ICAM1* | ATGCCCAGACATCTGTGTCC | GGGGTCTCTATGCCCAACAA |
| circ-CBLB | GAGACAAGTAAGACTGTGCCAAA | TGGGCAAGTTTCTGGTTGTC |
| circ-ITPR3 | TGTTTGTGGTGAGCTTCGTG | CCAGGAACCAGGATGCTGTA |
| circ-NFKBIA | ACAAATGACTGCCCTATGATGAC | TCCATGTTCTTTCAGCCCCT |
| circ-ICAM1 | GCCCAGACTTCCTTTGTGTT | CATTATGACTGCGGCTGCTA |
| *GAPDH* | ACCCACTCCTCCACCTTTGAC | TGTTGCTGTAGCCAAATTCGTT |

Supplemental Table 2 Characteristics of the study participants

|  | GDM | Control | *t/χ^2^* | *P* |
| --- | --- | --- | --- | --- |
| Number | 34 | 34 |  |  |
| Maternal age (years) | 30.53±4.49 | 30.41±4.49 | -0.11 | 0.914 |
| Gestational age (weeks) | 38.32±1.25 | 38.54±1.07 | 0.69 | 0.494 |
| Multipara | 19(55.9%) | 19(55.9%) | 0.00 | 1.000 |
| Family history of diabetes^a^ | 2(5.9%) | 0(0.0%) |  | 0.493 |
| Pre-pregnancy BMI (kg/m^2^) | 22.76±3.94 | 23.05±3.86 | 0.31 | 0.761 |
| Pregnancy BMI (kg/m^2^) | 26.43±3.34 | 27.06±3.41 | 0.77 | 0.444 |
| Abdominal circumference (cm) | 99.50±6.01 | 101.00±5.89 | 1.04 | 0.302 |
| Systolic pressure (mmHg) | 110.09±11.20 | 111.24±11.10 | 0.42 | 0.673 |
| Diastolic pressure (mmHg) | 70.35±8.18 | 69.41±7.84 | -0.48 | 0.630 |
| Fasting blood glucose (mmol/L) | 4.75±0.42 | 4.32±0.29 | -4.55 | <0.001 |
| 1h OGTT (mmol/L) | 8.61±2.47 | 6.98±2.22 | -2.68 | 0.010 |
| 2h OGTT (mmol/L) | 8.07±1.41 | 6.70±0.88 | -4.41 | <0.001 |
| TG (mmol/L) | 4.27±2.27 | 4.40±2.99 | 0.21 | 0.833 |
| TC (mmol/L) | 6.43±1.01 | 6.71±1.60 | 0.89 | 0.377 |
| HDL-c (mmol/L) | 1.79±0.32 | 1.81±0.31 | 0.21 | 0.837 |
| LDL-c (mmol/L) | 3.61±0.57 | 3.82±0.80 | 1.28 | 0.206 |
| Cesarean section | 11(32.4%) | 17(50.0%) | 2.19 | 0.139 |
| Macrosomia^a^ | 3(9.1%) | 2(5.9%) |  | 0.673 |

^a^ Represents using the Fisher exact probability method to compare the differences between groups.

BMI: Body Mass Index, TG: Triglyceride, TC: Total cholesterol.

Supplemental Table 3 Top 10 circRNAs of up-regulation and down-regulation

| circRNA | regulation | fold change | *P* |
| --- | --- | --- | --- |
| hsa_circ_0056021 | up | 12.88 | 1.11E-06 |
| hsa-circRNA70-2 | up | 10.19 | 2.25E-06 |
| hsa_circ_0056022 | up | 4.88 | 3.21E-06 |
| hsa-circRNA70-3 | up | 5.60 | 3.30E-06 |
| hsa_circ_0056023 | up | 4.34 | 4.13E-06 |
| hsa-circRNA70-4 | up | 30.34 | 4.20E-06 |
| hsa_circ_0056024 | up | 5.37 | 4.72E-06 |
| hsa-circRNA70-5 | up | 6.69 | 5.20E-06 |
| hsa_circ_0056025 | up | 11.76 | 5.68E-06 |
| hsa-circRNA7 0-6 | up | 6.85 | 8.36E-06 |
| hsa_circ_0056031 | down | 4.45 | 1.65E-06 |
| hsa-circRNA70-12 | down | 3.37 | 5.60E-06 |
| hsa_circ_0056032 | down | 4.14 | 7.66E-06 |
| hsa-circRNA70-13 | down | 4.28 | 8.54E-06 |
| hsa_circ_0056033 | down | 3.48 | 1.11E-05 |
| hsa-circRNA70-14 | down | 6.26 | 1.92E-05 |
| hsa_circ_0056034 | down | 3.70 | 2.48E-05 |
| hsa-circRNA70-15 | down | 3.12 | 2.48E-05 |
| hsa_circ_0056035 | down | 6.20 | 2.51E-05 |
| hsa-circRNA70-16 | down | 2.89 | 2.79E-05 |

Supplemental Table 4 Top 10 mRNAs of up-regulation and down-regulation

| mRNA | regulation | fold change | *P* |
| --- | --- | --- | --- |
| *PASK* | up | 3.92 | 7.57E-05 |
| *CD79B* | up | 2.36 | 9.63E-05 |
| *SLAMF1* | up | 2.55 | 1.44E-04 |
| *CNR2* | up | 2.21 | 1.93E-04 |
| *FREM2* | up | 2.23 | 1.98E-04 |
| *BEND5* | up | 2.49 | 2.00E-04 |
| *NMNAT2* | up | 2.32 | 2.23E-04 |
| *CCR4* | up | 2.73 | 2.30E-04 |
| *CD40LG* | up | 3.44 | 2.40E-04 |
| *YPEL1* | up | 2.11 | 2.77E-04 |
| *IFNAR1* | down | 2.49 | 2.34E-04 |
| *SULT1B1* | down | 2.51 | 3.65E-04 |
| *LAMP2* | down | 2.19 | 4.54E-04 |
| *CSF2RB* | down | 2.90 | 4.71E-04 |
| *ABCB4* | down | 2.47 | 4.74E-04 |
| *LINC01350* | down | 2.17 | 4.83E-04 |
| *AVIL* | down | 4.93 | 5.14E-04 |
| *LPCAT2* | down | 2.63 | 5.70E-04 |
| *SLC30A1* | down | 2.19 | 6.06E-04 |
| *TET2* | down | 2.78 | 6.67E-04 |

Supplemental Table 5 Expression results of candidate mRNAs and circRNAs in microarrays

| name | circRNA ID | regulation | fold change | *P* value |
| --- | --- | --- | --- | --- |
| *CBLB* |  | up | 2.02 | 0.014 |
| *ITPR3* |  | up | 2.35 | 0.009 |
| *NFKBIA* |  | up | 3.57 | 0.021 |
| *ICAM1* |  | up | 2.92 | 0.005 |
| circ-CBLB | hsa_circ_0121399 | up | 2.31 | 0.032 |
| circ-ITPR3 | hsa_circ_0076000 | up | 2.22 | 0.022 |
| circ-NFKBIA | hsa_circ_0031660 | up | 2.57 | 0.009 |
| circ-ICAM1 | hsa_circ_0049241 | up | 3.52 | 0.002 |
